# The measurement of erythrocyte transketolase activity on a discrete analyser

**DOI:** 10.1155/S1463924682000455

**Published:** 1982

**Authors:** C. R. Milner, J. E. Buttery, B. R. Chamberlain

**Affiliations:** Department of Clinical Chemistry The Queen Elizabeth Hospital Woodville South Australia 5011 Australia


Journal of Automatic Chemistry, Volume 4, Number 4 (October–December 1982), pages 183–185

### The measurement of erythrocyte transketolase activity on a discrete analyser

**C. R. Milner, J. E. Buttery and B. R. Chamberlain**

*Department of Clinical Chemistry, The Queen Elizabeth Hospital, Woodville, South Australia 5011, Australia*

Transketolase (TK, EC.2.2.1.1) is a thiamine-dependent enzyme which is widely used for assessing thiamine deficiency. The TK activity in erythrocytes is usually decreased during thiamine deficiency and this activity can be enhanced *in vitro* by the addition of thiamine pyrophosphate (TPP). The relative increase in enzyme activity, expressed as a percentage, is called the TPP effect.

The TK kinetic method proposed by Smeets *et al.* [1] is relatively simple and specific and is based on the measurement of NADH decrease. Minor modifications of this method have been proposed [2 and 3] but the method remains tedious if several specimens are assayed. To further simplify the assay, we have modified the method of Smeets *et al.* for a discrete enzyme analyser, the Gilford System 5 (Gilford Instrument Laboratories Inc., Oberlin, Ohio, USA) to give a very simple, economical and convenient method.

### Method

### *Reagents*

These are essentially similar to those of Smeets *et al.*, but the preparation of reagents modified to improve their storage is outlined.

(1) Tris buffer, 0·1 mol/l, pH 7·8 at room temperature [RT]. Dissolve 10·64 g Trizma HCl (Sigma) and 3·94 g Trizma base (Sigma) in 1·0 litres of water. Store at RT.
(2) Thiamine pyrophosphate, 0·01 mol/l in tris buffer. Dissolve 46·1 mg TPP (Sigma) in 10 ml buffer. Store 1·0 ml portions at −20°C.
(3) Ribose-5-phosphate, 37·5 mmol/l in tris buffer. Dissolve 1 g sodium ribose-5-phosphate (Sigma) in 91 ml buffer. Store at −20°C in 5 or 10 ml portions (unused portions can be refrozen and reused).
(4) NADH, 0·01 mol/l in tris buffer. Dissolve 7·46 mg $NADH.Na_2$ (Sigma) in 1·0 ml buffer. Prepare fresh the approximate amount required.
(5) Glycerol-3-phosphate dehydrogenase/triosephosphate isomerase (GDH/TIM; 2 mg/ml) in ammonium sulphate solution (Boehringer). Store at 4°C.

### *Sample preparation*

Blood is collected in a heparinized tube and centrifuged as soon as possible. The plasma and buffy coat are removed and the erythrocytes washed in cold physiological saline and again centrifuged. The packed erythrocytes are haemolysed by mixing with an equal volume of sterox solution 5 ml/l (Sterox SE, Harleco Company, Philadelphia, Pennsylvania 19143, USA), and stored at −20°C.

Just prior to the assay, the haemoglobin concentration is determined by the cyanmethaemoglobin method. This concentration is then adjusted to 30 g/l with saline and centrifuged to obtain a haemolysate free of stroma.

### *Gilford System 5*

This is a discrete analyser which consists of a spectrophotometer with a flow-through thermocuvette, computer/printer, automatic dispenser and specimen transport. There are several operating modes including kinetic pre-programmed and general modes, making the analyser versatile and simple to use.

In the kinetic mode the specimen is placed into cups in the transport carousel. The reagent is placed into the automatic dispenser and the computer is programmed to the requirements of the assay. When in operation, the required volume of reagent is dispensed into the sample cup. After the appropriate lag phase the reaction mixture is aspirated into the thermocuvette where equilibrium is reached, then absorbances are read over a predetermined time and the enzyme activity calculated and printed.

### *Reagent mixture*

The proportions given below are sufficient for nine tests, namely four specimens (TK and TPP effect for each) and one 'blank'.

| | |
|---|---|
| Ribose-5-P substrate: | 5 ml |
| Tris buffer: | 5 ml |
| NADH solution: | 250 $\mu$l |
| GDH/TIM suspension: | 50 $\mu$l. |

The reagent mixture is then placed in the dispenser bottle set to deliver 1·0 ml. A proportional quantity of reagent is prepared depending on the number of tests.

### *Sample loading*

Adjacent pairs of sample cups are used for the TK and TK-TPP test for each patient sample, and these contain 0·5 ml buffer, and 0·45 ml buffer and 50 $\mu$l TPP respectively.

A precision pipette is used to introduce 50 $\mu$l haemolysate into each pair of sample cups, using the 'wash-out' technique to transfer the haemolysate. This is then stirred with the pipette tip to achieve even mixing of the reagent and haemolysate. Failure to do this sometimes causes erroneous results.

A sample cup containing 0·5 ml buffer and 50 $\mu$l haemolysate is used as the blank and is placed in front of the first test sample. This blank serves only to prime the Gilford System and its result is not used.

### *Operational mode*

The initial absorbance of the Gilford spectrophotometer is adjusted to about −0·8 with water.

The 'enzyme-general mode', test number 25, is used to program the Gilford computer/printer. The parameters for the TK assay are:

| | |
|---|---|
| Wavelength: | 340 nm |
| Temperature: | 37°C |

0142-0453/82/0404 0183 $05·00 © 1982 Taylor & Francis Ltd

| | |
|---|---|
| Lag phase time: | 1600 s |
| Equilibrium time: | 300 s |
| Read time: | 120 s |
| Factor: | −166. |

After verifying that the parameters are correct and that the sample tray is properly positioned, the test is initiated.

### Results

### *Comparison between two methods*

Both methods showed good correlation for the TK assay (figure 1) and for the activated TK assay (figure 2). Additional analytical data on the two tests are given in table 1.

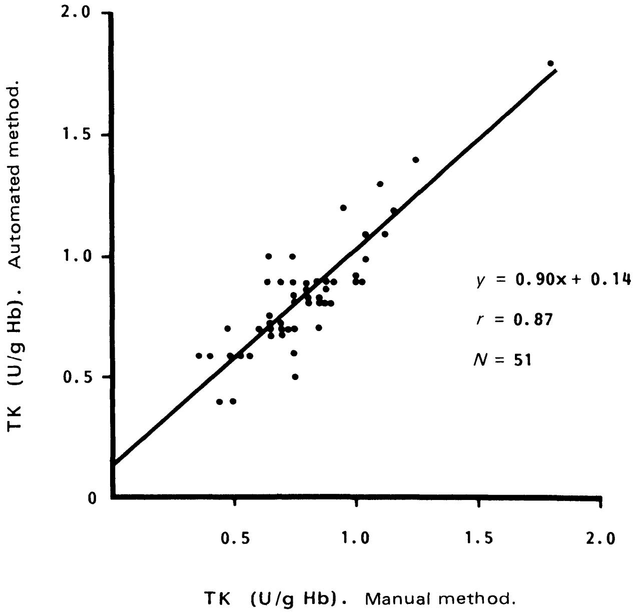

*Figure 1. Correlation between transketolase automated method and the manual method.*

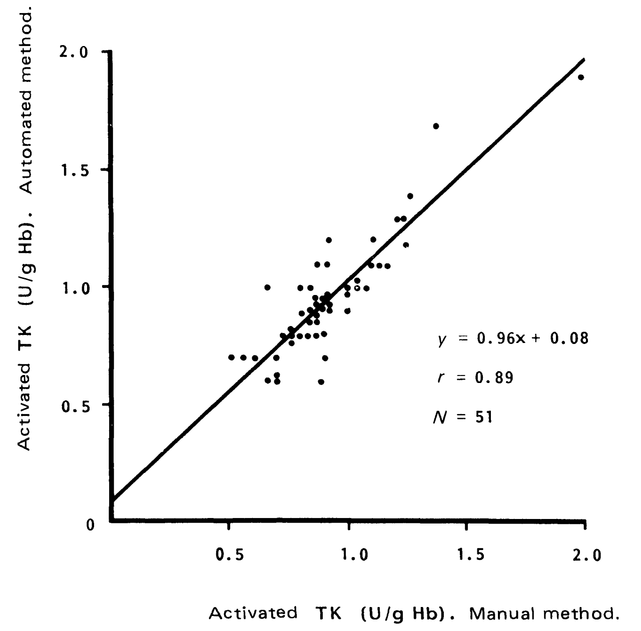

*Figure 2. Correlation between thiamine activated transketolase automated method and the manual method.*

*Table 1. Analytical details of TK activities determined by the two methods.*

| | TK | | TK (+TPP) | |
|---|---|---|---|---|
| | Manual | Gilford | Manual | Gilford |
| Mean (U/g Hb) | 0·81 | 0·86 | 0·94 | 0·98 |
| SD (U/g Hb) | 0·25 | 0·26 | 0·25 | 0·27 |
| Range | 0·31–1·31 | 0·34–1·38 | 0·44–1·44 | 0·44–1·52 |
| *N* | 51 | 51 | 51 | 51 |

### *Accuracy in measurement*

Accuracy was further determined by assaying two specimens having known low (L) and high (H) TK activities and three specimens prepared in the following proportions: (2L + 1H), (H + L), (1L + 2H). Figure 3 shows the TK activities of the above samples and the expected correlation line. It would appear that our modified method on the Gilford analyser is quite accurate.

### *Precision*

The within-day precision determined for the modified Gilford method on samples with 'low' and 'high normal' TK activities is shown in table 2. The day-to-day precision has not been done owing to specimen instability.

*Table 2. Within-day precision determined for the modified Gilford method.*

| | Within-day precision | |
|---|---|---|
| | 'Low' TK | 'High normal' TK |
| Mean (U/g Hb) | 0·41 | 1·05 |
| SD (U/g Hb) | 0·03 | 0·05 |
| CV (%) | 7·4 | 5·0 |
| *N* | 11 | 10 |

### *Stability of samples during assay*

The time taken to assay five specimens (10 tests and one blank) is about 100 min. The stability of samples over this period was assessed on several occasions by placing them at the beginning and at the end of the assay: no loss of enzyme activity was detected. However, delay in excess of 2 h causes a fall in enzyme activity of 0·1 U/g Hb. This occurs when large batch samples are loaded on the sample tray at the same time.

### *TPP effect*

The upper limit of the TPP effect is normally taken to be 25%. From the 51 patients randomly selected for this study, five showed elevated (abnormal) TPP effect by the manual method and only three were abnormal by the automated method. Of the two patients who showed equivocal results, one had a normal TK activity by both methods, but the other had a low TK and TPP effect by the automated method. We were unable to repeat the latter observations to confirm the finding.

### Discussion

A semi-automated TK method adapted to the centrifugal fast analyser based on the measurement of glucose-6-phosphate has been proposed [4]. The method of Smeets *et al.* has, to our knowledge, not been automated. A disadvantage of this method

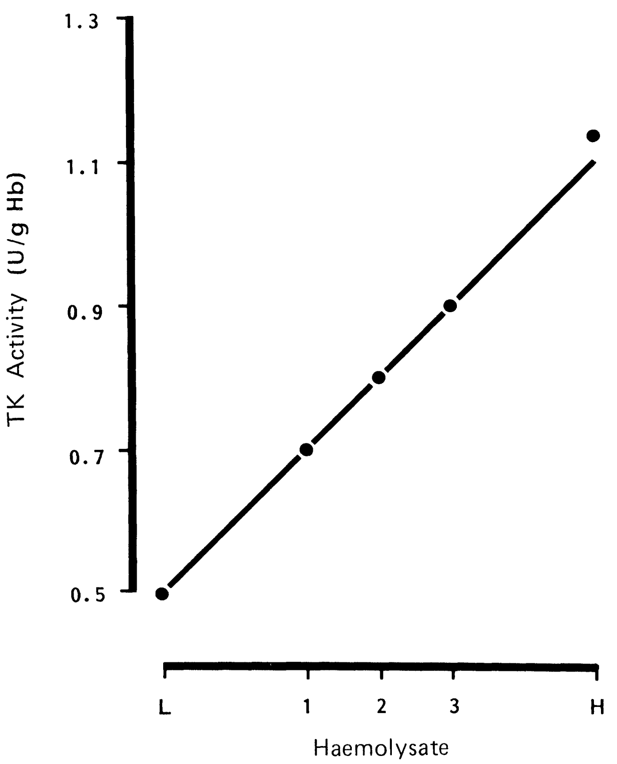

*Figure 3. Transketolase activities determined from haemolysates prepared in varying proportions.*

when performed manually is that it is very demanding on the analyst's time. With a suitable spectrophotometer, such as the Unicam SP8000, four tests can be performed in an analytical run, but despite this the change-over of tests and the calculation of enzyme activity is time-consuming. This is not so for our modified TK method on the Gilford analyser. Specimens can be loaded in the sample cups and left unattended until the test is completed. There is no loss in enzyme activity when specimens are left in the cups for up to 100 min before analysis. If this time is markedly exceeded there is some loss in enzyme activity. Thus for a large batch of say 10 specimens (i.e. 20 tests), we recommend that the specimens be loaded in two or more stages so that they are not left for an unduly long time. To minimize or prevent enzyme deterioration, specimens waiting to be assayed should be stored in a refrigerator.

For the automated assay the optimum lag time, established mainly at room temperature which will allow the TK reaction to proceed at a constant rate, similar to the manual method, was found to be 1600 s, of which the last 300 s is at 37°C.

The absorbance of the TK test is high and this is due to the NADH and the haemoglobin present. Only instruments which can offset some of this initial high absorbance will be able to measure the decreasing NADH absorbance change due to the TK assay. Both the Unicam SP8000 and the Gilford spectrophotometers can do this adequately.

Extrapolated from our previous data [5], the range for the TK activity was 0·6 to 1·3 U/g Hb, while the range for the thiamine pyrophosphate (TPP) effect was 0–25%.

### Acknowledgement

The authors would like to thank Dr M. L. Wellby for his interest and comments.

### References

1. Smeets, E. H. J., Muller, H. and De Wael, J., *Clinica Chimica Acta*, **33** (1971), 379.
2. Williams, D. G., *Clinical Biochemistry*, **9** (1976), 252.
3. Bayoumi, R. A. and Rosalki, S. B., *Clinical Chemistry*, **22** (1976), 327.
4. Van Zanten, A. P., Beijer, C., Mairuhu, W. M. and Van Den Ende, A., *Clinica Chimica Acta*, **105** (1980), 303.
5. Buttery, J. E., Milner, C. R. and Chamberlain, B. R., *Clinica Chimica Acta*, **102** (1980), 221.

### CONFERENCE ANNOUNCEMENT

*VIIth International Symposium on Column Liquid Chromatography (2–6 May 1983)*

This is the seventh in a series of successful meetings dealing with the latest developments in column liquid chromatography. The scientific programme will comprise discussion papers and poster communications covering all aspects of the subject and its related techniques. Lectures will be given in English.

The organizers would like to receive suggestions for papers, in the form of a 300-word abstract, by 15 November 1982. All submitted manscripts will be refereed by the scientific committee.

Those attending the symposium will receive a booklet containing abstracts of the discussion papers and poster communications. Following the symposium, the papers will be published in a special edition of the *Journal of Chromatography.*

The VIIth International Symposium on Column Liquid Chromatography is sponsored by the Arbeitskreis Chromatographie der Fachgruppe Analytische Chemie in der Gesellschaft Deutscher Chemiker. Co-sponsors include the Chromatography Forum of the Delaware Valley, Groupement Pour l'Avancement Des Méthodes Spectroscopiques (GAMS), Koninklijke Nederlandse Chemische Vereniging, Schweizerischer Chemiker-Verband, Société Chimique de France, Svenska Kemistsamfundet and the Verein Österreichischer Chemiker.

An exhibition of liquid chromatography instruments and accessories will run concurrently with the meeting.

*Further details from Dr J. Wendenburg, c/o Gesellschaft Deutscher Chemiker, Varrentrappstrasse 40–42, D 6000 Frankfurt 90, FR Germany.*

